# The dual role of interleukin-6 in Crohn’s disease pathophysiology

**DOI:** 10.3389/fimmu.2023.1295230

**Published:** 2023-12-01

**Authors:** Ala’ Alhendi, Saleh A. Naser

**Affiliations:** Division of Molecular Microbiology, Burnett School of Biomedical Sciences, College of Medicine, University of Central Florida, Orlando, FL, United States

**Keywords:** IL-6, MAP, paratuberculosis, Crohn’s disease, therapeutic targets

## Abstract

Interleukin-6 (IL-6) is a key mediator cytokine of the immune response as well as a regulator of many physiological and pathological processes. In Crohn’s disease (CD), cytokine imbalance rules the intestinal microenvironment and leads to chronic inflammation of the gut. Pro-inflammatory cytokines are generally upregulated in inflammatory bowel disease (IBD) including TNFα and IL-6. Consequently, drugs that target these cytokines have been long sought and approved. Despite the short-term success in treating CD patients with anti-TNFα, many patients stopped responding to treatment, which made IL-6 an alternative target to alleviate inflammation in these patients. IL-6 has long been approached as part of the therapeutic strategies to treat CD and other inflammatory disorders. Clinical trials of CD patients have targeted IL-6 signaling in different mechanisms: blocking IL-6, neutralizing IL-6 receptor (IL-6R), or trapping the soluble IL-6/IL-6R complex. These trials have faced challenges and side effects in patients with gastrointestinal perforations and ulcers, for example, all of which highlight the dual role of IL-6 during intestinal inflammation and the need for this cytokine for intestinal tissue integrity. IL-6 is involved in a complex of upstream regulators and downstream signaling cascades and maintaining a physiological level of IL-6 in the blood and in the intestine is key for achieving health and homeostasis. In this review, we describe IL-6 biology and signaling and its involvement in intestinal health and inflammation. We also discuss the current strategies for targeting IL-6 pathways in CD patients, as well as molecular regulators representing potential therapeutic targets for IL-6 attenuation.

## Introduction

Inflammatory bowel disease (IBD) is a group of debilitating chronic inflammatory intestinal disorders that consists of ulcerative colitis (UC) and Crohn’s disease (CD), where inflammation dynamics differ between the two subtypes (1). CD is characterized by intermittent flares and repeated remissions. Flares are usually characterized by moderate to severe inflammatory episodes of debilitating symptoms, which then progress to cause intestinal epithelial damage and mucosal dysfunction leading to serious complications like strictures, fistulas, and ulcer formation. Multiple genetic and environmental factors play a role in the pathogenesis of CD (2, 3). Regardless of the underlying cause of CD, pathophysiology in CD patients is ruled by the loss of balance between pro-inflammatory and anti-inflammatory cytokines secreted primarily by Th1/Th17 helper T cells and regulatory T cells, respectively. The upregulated pro-inflammatory cytokines include IFNγ, TNFα, IL-6, and IL-1. In contrast, inflammation-resolving cytokines such as IL-10 and TGFβ are often downregulated (1, 4).

Among key inflammatory cytokines involved in CD, IL-6 is a significant contributor to the inflammation and pathogenesis in IBD (5). IL-6 production and signaling are upregulated in inflamed mucosa and plasma of IBD patients, and the increase is more pronounced in patients with CD compared to UC patients (6, 7). While the IL-6 physiological plasma level is approximately 1.6 pg/ml, it can increase up to 32.7 ng/ml in CD patients (6, 8). IL-6 excessive production in IBD patients originates from peripheral blood mononuclear cells (PBMCs) and intestinal lamina propria mononuclear cells (LPMCs) (9, 10). Generally, IL-6 is secreted by intestinal epithelia, intestinal smooth muscle cells, CD4^+^ T cells, and macrophages (8, 11). IL-6 is essential in sustaining a chronic inflammation in IBD mainly by promoting CD4 T-cell resistance to apoptosis. This is achieved by a STAT3-dependent upregulation of anti-apoptotic proteins bcl-2 and bcl-xL in T cells, promoting their longevity and the consequent perpetuation of chronic inflammation (12, 13).

IL-6 is a versatile cytokine that plays a pivotal role in the shift between intestinal homeostasis and inflammation. In the gastrointestinal tract, IL-6 exhibits diverse functions that range from maintaining the intestinal epithelium integrity and mucosal barrier function to the modulation of immune response against environmental microbes (8).

## The biology of IL-6

**Figure 1:**
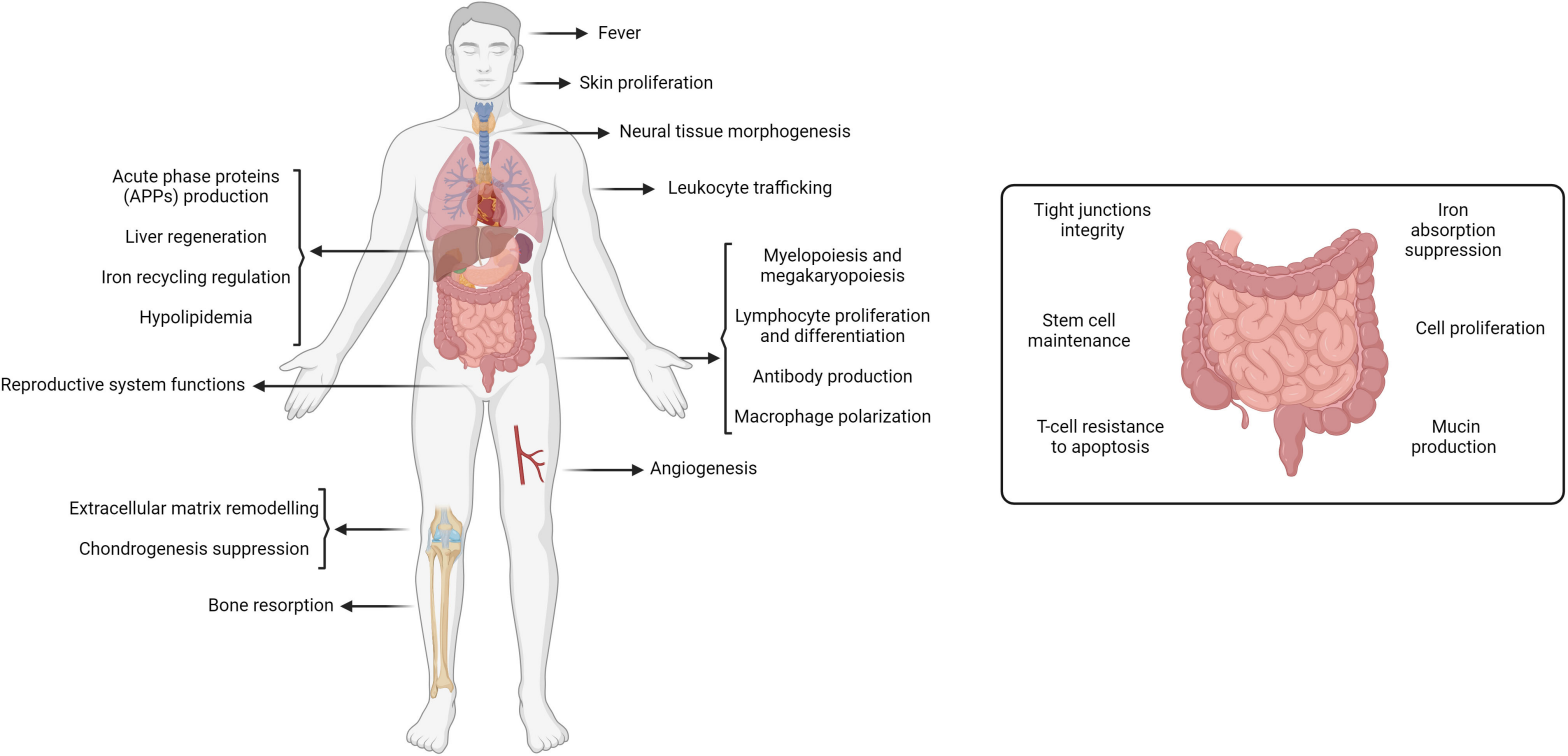
Physiological role of IL-6. IL-6 plays an essential role in most tissues including the liver, immune cells, skin, neural tissue, reproductive system, blood vessels, bone, cartilage, and intestines. Generated by Biorender.com.

IL-6 was first discovered as a soluble factor secreted by helper T cells to induce antibody production and release by B cells. It was originally termed B-cell stimulatory factor-2 (BSF-2) or B-cell differentiation factor (BCDF) (14). IL-6 is a pleiotropic cytokine that regulates diverse tissues and physiological processes (**Figure 1**). It plays a crucial role in immune functions during various stages, from mediating myelopoiesis to activating and differentiating immune cells like macrophages, T cells, and B cells while also facilitating the trafficking of neutrophils to sites of infection. In the liver, IL-6 is needed for tissue regeneration after injury and acute phase protein production during inflammation. Moreover, IL-6 induces fever during infection, activates VEGF-mediated angiogenesis, modulates bone and cartilage remodeling, plays a role in neural cell proliferation and differentiation, and regulates iron and lipid metabolism (5, 11, 15, 16).

### IL-6 signaling

IL-6 functions through specific receptors located on target cells. IL-6 receptor (IL-6R) is limited to hemopoietic cells, hepatocytes, and some epithelial cells (11, 17). IL-6R is found bound to cell membranes of these particular cells or secreted in a soluble form in biological fluids. The soluble form of the receptor can be generated from the enzymatic cleavage of the membrane-anchored form by ADAM-17 metalloproteinase (also known as TNFα-cleavage enzyme (TACE)) or by the alternative splicing of IL-6R mRNA (18).

**Figure 2:**
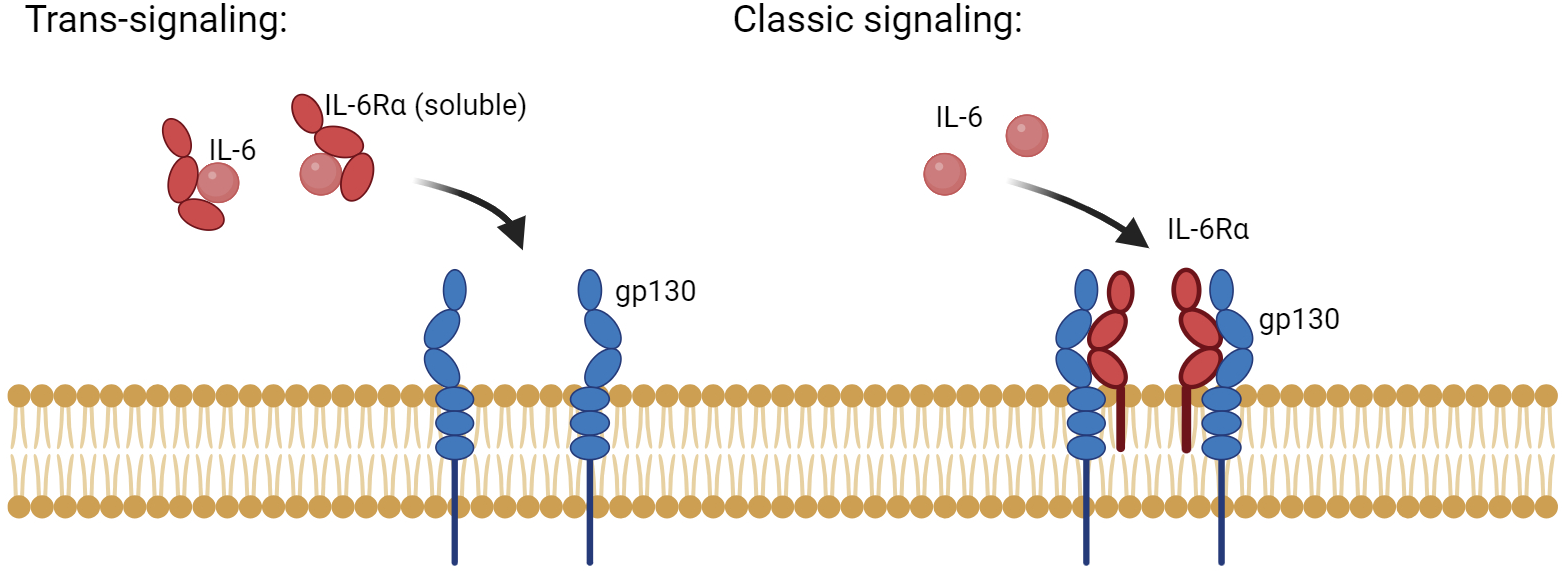
The two main modes of IL-6 signaling: classic and trans-signaling. Classic IL-6 signaling affects a limited number of cells that inherently express the membrane-anchored form of IL-6R and the signaling component, gp130. In contrast, trans-signaling affects cells that lack a membrane expression of IL-6R but have gp130. Both modes of signaling require the presence of a hexamer of two of each: IL-6, IL-6R, and gp130. Generated by Biorender.com.

**Figure 3:**
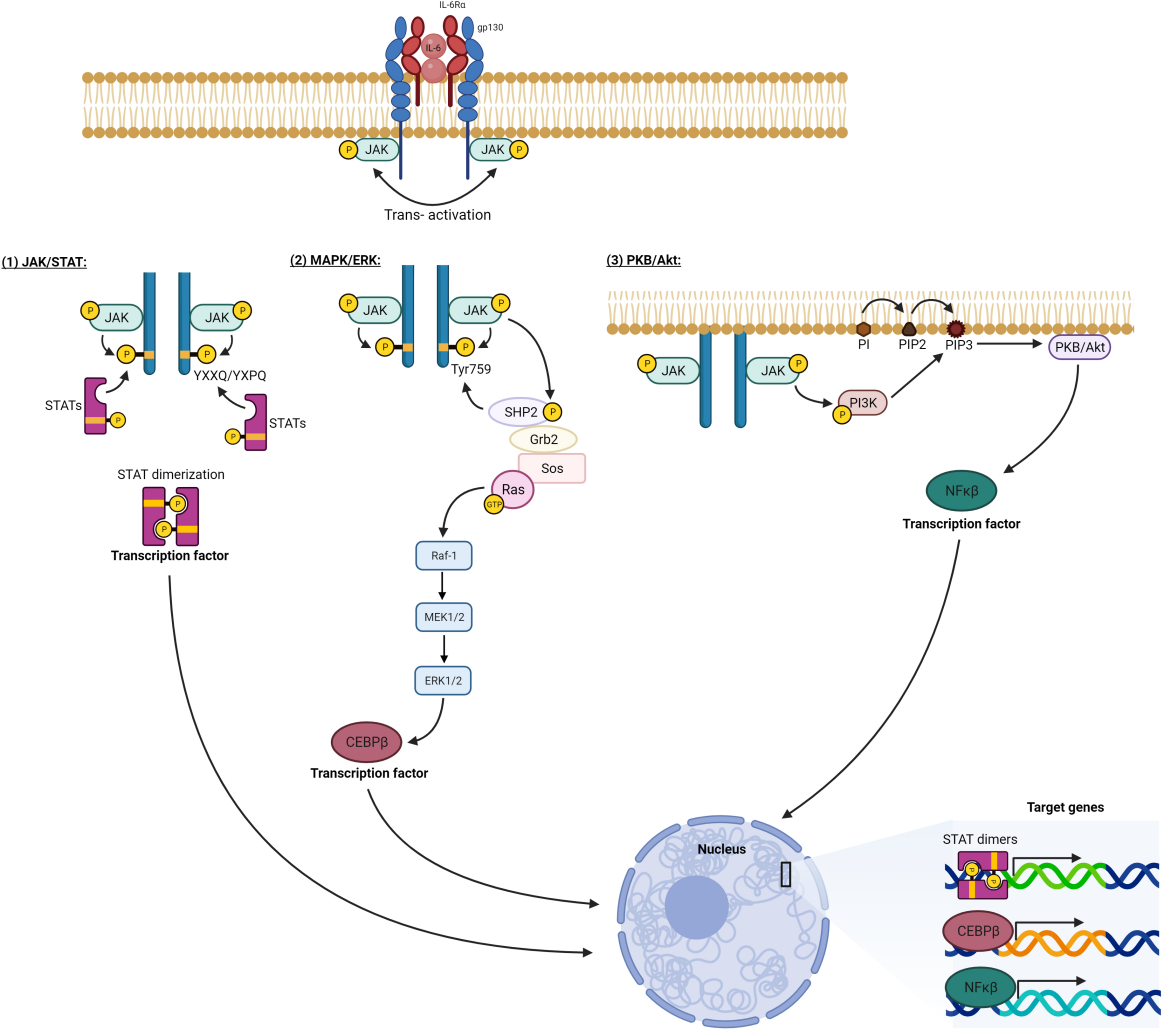
IL-6 intracellular signaling pathways. Activation of the hexameric IL-6 signaling complex brings the cytoplasmic tails of gp130 into proximity. This allows the adjoined JAKs to transphosphorylate each other as well as tyrosine residues on the gp130 cytoplasmic portion. 1) Phosphorylated tyrosines at YXXQ/YXPQ sequences act as docking sites for STAT transcription factors. These STATs are consequently phosphorylated by the activated JAKs leading to their dimerization and translocation to the nucleus. 2) Phosphorylated TYR759 on gp130 recruits a cascade of signaling mediators, which eventually activate Ras protein initiating the MAPK pathway (Raf/MEK/ERK), which eventually activates the transcription factor CEBPβ. 3) Activated JAK phosphorylates and activates PI3K, which generates PIP3 on the plasma membrane and docks for PKB/Akt, which is phosphorylated and activated, initiating multiple downstream pathways, one of which activates transcription factor NFκB. All the activated transcription factors translocate to the nucleus, and by binding. DNA response elements modulate the expression of target genes. Generated by Biorender.com.

Activation of IL-6 signaling requires the binding of IL-6 with IL-6R (α-receptor) and glycoprotein 130 (gp130, also known as the β-receptor) (19). The functional complex of IL-6/IL-6R/gp130 has been illustrated using X-ray crystallography to be a hexamer of two proteins of each of IL-6, IL-6R, and gp130. The congregation of this hexameric complex activates gp130-associated Janus kinases (JAKs). JAKs in turn phosphorylate tyrosine residues on the cytoplasmic tail of gp130 and recruit STATs (Signal Transducer and Activator of Transcription) to specified phosphorylated residues. JAKs proceed to phosphorylate and activate these STATs, which then dimerize and translocate into the nucleus to modulate gene expression of target genes (11). IL-6 signaling is either classic *via* membrane-bound IL-6R or trans *via* soluble IL-6R (**Figure 2**) (20). While classic signaling affects a limited variety of cells, trans-signaling is almost universal, affecting all gp130-expressing cells. The ubiquitous distribution of gp130 around the body allows a larger group of IL-6-responsive cells (21). Trans-signaling, *via* the circulating IL-6 and its receptor, is believed to be the reason for the chronic inflammation effect of IL-6. It was deemed responsible for activating cells that lack a membrane-bound IL-6R like endothelial cells to produce chemokines needed for the recruitment of mononuclear immune cells. Recruitment of these cells *via* trans-signaling and not through the classic pathway was specifically responsible for inflammation in the air-pouch model of inflammation in mice (22). In addition to the JAK/STAT signaling axis, both classic and trans-signaling modes activate other intracellular pathways like MAPK/ERK and PKB/AKT as demonstrated in **Figure 3** (23).

## IL-6 signaling in intestinal homeostasis

As shown earlier in **Figure 1**, IL-6 leads to specialized functions in the intestine, which are essential for maintaining intestinal barrier integrity, microbial defense, immune homeostasis, and cell differentiation and survival.

Under physiological conditions, IL-6 is needed for mucin production and to maintain a balanced intestinal epithelial permeability. Gut microbiota stimulates the intraepithelial lymphocytes (IELs) to release IL-6, which is needed to maintain the mucosal barrier integrity. This was proven by the disrupted tight junctions, paracellular permeability, and mucous layer seen in the intestines of IL-6^−/−^ mice. The absence of IL-6 in mice led to thinner intestinal mucous layers and increased intestinal paracellular permeability accompanied by reduced expression of tight junction proteins like claudin-1. IL-6, acting through the activation of STAT3, increases the expression of claudin-1 and MUC-2 *in vitro*. It is alternatively suggested that IL-6 can be protective in an indirect way by supporting the elimination of microbes and restoring the health of intestinal cells, making claudin-1 and MUC-2 more available (24).

Moreover, IL-6 is essential for epithelial cell proliferation and maintenance of the stem cell niche, which is needed for wound healing after intestinal injury (25). Blocking IL-6R or neutralizing IL-6 in the intestine reduces the stem cell population, cell proliferation, and crypt budding. This autocrine IL-6 activity in the intestinal crypts was achieved *via* the STAT3/Wnt signaling axis, which regulates crypt homeostasis (26).

## IL-6 signaling during inflammation and intestinal dysfunction and restoration

IL-6 contributes to the damage of the intestinal epithelial barrier integrity during inflammatory conditions by increasing intestinal permeability. Claudin-2, a tight junction protein responsible for increasing the permeability of epithelial layers to small ions (cations), is a well-established marker of intestinal permeability and a direct target of IL-6 signaling. Functionally, claudin-2 expression is reflected in the electrical resistance of the epithelial barrier indicating its permeability to cations (27). IL-6 damage to the intestinal barrier is manifested by upregulation of claudin-2 expression, reduction in the transepithelial electrical resistance (TEER), and increased intestinal epithelial layer permeability to small molecules like urea in cultured Caco-2 cells (28, 29). Clinically, upregulated claudin-2 is a predominant feature of the inflamed intestinal mucosa of CD patients and participates in leak flux (exudative) diarrhea, which is also seen in patients with ulcerative colitis (27, 30). Analysis of colonic biopsies from mild to moderate CD patients proved an impairment of tight junctions and increased apoptosis in CD compared to controls. This was in conjunction with the upregulated leaky tight junction protein, claudin-2 (31). Interestingly, the upregulation of claudin-2 by IL-6 was achieved by different signaling pathways in two separate studies, with the final result being the same. In one study, claudin-2 expression was upregulated *via* MAPK/ERK and PI3K/AKT pathways and with the action of the transcription factor Cdx2 (28). Claudin-2 expression also was dependent on JNK kinase activation and the subsequent phosphorylation of the transcription factor AP-1, which binds onto the claudin-2 promoter (29). Other tight junction proteins like occludin and claudin-1 are also modulated by IL-6 as has been demonstrated in a rat model of chronic stress where IL-6 levels increase consequentially. The spike in IL-6 is accompanied by a decrease in these junctional proteins and a following increase in the intestinal paracellular permeability. These changes are attributed to the increase of methylation at the H3K9 histone site of both occludin and claudin-1 promoters and the reduction in glucocorticoid receptor (GR) binding to these sites. *In vitro* treatment of Caco-2/BBE cells with IL-6 also decreased occludin and claudin-1 expression, and this was reversed when H3K9 methylation was specifically blocked using UNC0638 (a H3K9 methylation inhibitor) (32). Moreover, the intestines of IL-6-wild-type mice are more vulnerable to sepsis and have increased paracellular and transcellular transport in response compared to mice lacking IL-6 expression. This effect was a result of a crosstalk between IL-6 and other cytokines like IL-10 (33).

Furthermore, IL-6 is crucial for regulating the immune response within the intestinal microenvironment. For instance, IL-6 activates the pro-inflammatory NFκB signaling pathway in intestinal cells leading to upregulation of ICAM-1 expression (34). ICAM-1, an epithelial adhesion molecule, is required for neutrophil interactions with the epithelia during neutrophil trafficking and thus plays a role in inflammation (35). IL-6 trans-signaling *via* the soluble IL-6R renders T cells resistant to apoptosis. Isolation of lamina propria CD3^+^ T cells from CD patients showed higher expression of the anti-apoptotic proteins, bcl-2 and bcl-xl, in these cells compared to cells isolated from controls. This anti-apoptotic shift was found to be STAT3-dependent and allows T cells to persist longer during chronic inflammation. This is reversed by a neutralizing antibody against IL-6R as well as by the fusion protein gp130-Fc, both of which block IL-6 signaling. The latter specifically blocks trans-signaling *via* the soluble receptor but not the membrane-bound receptor (i.e., classical signaling). Both blocking mechanisms of IL-6 signaling lead to lamina propria T-cell death by apoptosis. Interestingly, the fusion protein (gp130-Fc), which specifically inhibits the trans-signaling pathway, induced T-cell apoptosis to an extent very similar to that of IL-6R blocking, indicating the prominent contribution of IL-6 trans-signaling on T-cell resistance to apoptosis during inflammation (13).

## Targeting IL-6 for CD therapy

Previous CD therapy focused on the non-specific inhibition of the immune system with the use of corticosteroids or immunosuppressive agents like thiopurines or methotrexate. The use of corticosteroids reduces CD mortality but involves side effects that halt its long-term use. Current therapeutic approaches vary between corticosteroids, immunosuppressive agents, antibiotics, and biological therapies that target either cytokines like TNFα, IL-12/IL-23, or integrins like α4β7 and surgical resection (36). IL-6 imposes an attractive target for treatment in IBD, as it is further involved in the pathogenesis of colorectal cancer. Targeting IL-6 can thus relieve inflammation in the gastrointestinal tract and reduce the risk of carcinogenesis (5). Different strategies have been explored to block IL-6 signaling for the treatment of IBD. We mention here the most prominent ones in the journey to control IL-6 signaling.

Because IL-6R has less inter-patient level variability compared to IL-6, it has been selected earlier for therapeutic targeting. A humanized monoclonal antibody (tocilizumab, initially known as MRA) against IL-6R has been produced and used in the treatment of multiple autoimmune and inflammatory diseases including rheumatoid arthritis (RA) and CD (19). Tocilizumab (TCZ) treatment of CD patients improved the disease activity index score in 80% of patients compared to 31% in the placebo group. Of TCZ-receiving patients, 20% went into remission compared to 0% in the placebo group (37). Unlike infliximab (anti-TNFα antibody), endoscopic and histologic healing was not observed in patients treated with TCZ. Few patients on TCZ suffered from gastrointestinal bleeding due to treatment, but a causative link with IL-6 blockade was not confirmed. Trials with TCZ were halted because of intestinal perforations found in rheumatoid arthritis clinical trials. It is noteworthy that most events of intestinal perforations occurred in patients with a pre-existing risk factor like intestinal diverticulitis or ulcers or patients who have used oral glucocorticoids (19).

In a phase II clinical trial, a fully human monoclonal immunoglobulin G2 against human IL-6 (PF-04236921) was used in CD patients. The study recruited 247 CD patients with unsatisfactory response to anti-TNFα therapy, and they were randomized in a double-blinded trial to one of three groups: placebo, 10 mg, or 50 mg of PF-04236921. The study met the established end points of improved CD activity parameters, and drug response and remission rates were significantly greater in the 50 mg treatment arm compared to other groups. Unfortunately, a proportion of patients treated with the antibody experienced gastrointestinal abscesses and perforations, as was reported previously in rheumatoid arthritis patients treated with TCZ (38).

It is worth mentioning that multiple studies of larger sample sizes contradict a higher risk of gastrointestinal (GI) complications in patients treated with TCZ compared to other conventional treatments of inflammatory diseases like CD and RA. A case study of a large rheumatoid arthritis patient population analyzed the frequency of serious adverse events in patients using TCZ (65,000 patient-years) in comparison to anti-TNFα (50,000 patient-years) therapies. This study included a bigger sample size compared to clinical trials by using different-sourced datasets of patients who used TCZ after the drug was marketed and available to the public. The reporting rate of serious gastrointestinal perforations in TCZ patients was found comparable to that of anti-TNFα patients. These results support the claim that TCZ does not impose a higher risk of serious adverse events of interest like GI perforations in the real-world setting when larger numbers of patients are assessed and included, in comparison to anti-TNFα therapies, which are commonly used (39). Before disease-modifying anti-rheumatic drugs (DMARDs) like anti-TNFα and TCZ were used, RA patients were treated with corticosteroids, which caused gastrointestinal complications and related deaths. In a systematic review comparing GI perforations in RA patients treated with corticosteroids, anti-TNFα, and TCZ, the reported rate of GI perforations of TCZ was within the range of that found in patients treated with corticosteroids or anti-TNFα. This could indicate that TCZ should not be singled out among CD therapies as the cause of GI perforations, and more strategies should be considered for treating patients of autoimmune diseases with IL-6 blockers before such a conclusion can be drawn (40).

A more recent approach to target IL-6 in IBD was to target the trans-signaling axis specifically. IL-6 classical signaling is thought to induce defensive inflammatory responses, while trans-signaling is involved in pathogenic chronic inflammation seen in IBD. To specifically target IL-6 trans-signaling, a decoy protein has been developed that is made of two gp130 extracellular domains fused to the Fc part of human IgG1 and is known as sgp130Fc or Olamkicept. In a phase 2a clinical trial that recruited 16 active IBD patients, 44% of patients showed clinical response, and 19% reached clinical remission. None of the patients reported intestinal perforations or severe adverse effects related to immunosuppression due to treatment. Olamkicept had strongly suppressed the STAT3 signaling pathway and more strongly in patients achieving clinical remission as was shown by transcriptomic analysis of patients’ intestinal mucosal biopsies. Olamkicept had no negative effect on epithelial cells’ proliferative or wound-healing abilities. Olamkicept, thus far, represents an outstanding drug with promising potential for the treatment of IBD by specifically targeting the IL-6 trans-signaling pathway (41).

## Intestinal regulators of IL-6 signaling as potential novel therapeutic targets

IL-6 signaling is subject to tight regulation by an intricate network of upstream molecular factors. The critical interplay between these molecular regulators ensures the precise and balanced activation of IL-6 signaling, which is essential for maintaining immune homeostasis and preventing excessive inflammation. Understanding the molecular regulators of IL-6 signaling holds significant therapeutic potential in the context of CD as well as other inflammatory disorders.

### TGFβ and serotonin

TGFβ is a well-studied upstream antagonist of IL-6 signaling in intestinal cells, which activates downstream Smad2 and Smad4, which translocate to the nucleus to activate target genes. Active Smad2/4 interferes with IL-6-induced phosphorylation of STAT3, which prevents the expression of ICAM-1, normally induced by active IL-6 signaling. ICAM-1, in contrast, is needed for IL-6 to induce leukocyte adhesion to the epithelium and endothelium, which secures their trafficking to intestinal tissue (42).

Another molecular regulator that connects to TGFβ is the monoamine neurotransmitter, serotonin (also known as 5-hydroxytryptamine (5-HT)). Activation of the serotonin receptor, 5-HT2B, protects against the initiation of colitis-associated cancer (CAC) *via* the canonical TGFβ/SMAD pathway, which inhibits the IL-6/STAT3 signaling pathway. 5-HT has a reputation for dual regulation in the context of intestinal inflammation and associated cancer. 5-HT/5-HT2B/TGFβ axis acts as a tumor suppressor in the initiation of CAC but then becomes a promoter of the established CAC at later stages. This shift occurs *via* the alternative activation of the non-canonical effector molecule of TGFβ signaling, Akt. Deletion of 5-HT2B from the mouse model of chemically induced colitis leads to an increased level of IL-6 and an accompanying upregulation of STAT3 activation, which boosts the survival and proliferation of intestinal epithelial cells during inflammation, leading to cancer initiation. The use of fluoxetine, a readily available anti-depressant drug and inhibitor of 5-HT reuptake, improved the weight of dextran sulfate sodium (DSS)-induced colitis mouse models (43). It may be valuable to consider repurposing this modulator for the treatment of early CD once clinical trials establish its efficacy in improving intestinal health under chronic inflammation in its early stages.

### Monocarboxylate transporter 4

Monocarboxylate transporter 4 (MCT4) is a cell-surface H^+^-coupled monocarboxylate symporter that was found to specifically upregulate IL-6 levels *in vivo* and *in vitro* (44, 45). MCT4 is expressed highly in hypoxic sites like metastatic tumors and inflammatory foci where lactate is involved in the modulation of immune responses. MCT4 has a high affinity for lactate and exports it against the concentration gradient (45). MCT4 is upregulated in the colons of IBD patients and DSS-induced experimental colitis mouse models. MCT4 activates the phosphorylation of p65 of NFκB, which translocates to the nucleus and interacts with the transcription coactivator CREB-binding protein (CBP), dissociating it from the CREB transcription complex. This activates the transcription of NFκB target genes comprising inflammatory cytokines like IL-1β, IL-6, and TNFα. Most notably, IL-6 was markedly increased in the serum of DSS animals and MCT4-transfected Caco-2 compared to other inflammatory cytokines. In contrast, MCT4 leads to a reduction in the transcriptional activity of CREB at the promoter of the tight junction protein, zonula occludens-1 (ZO-1). This leads to damaging the intestinal barrier function demonstrated by reduced TEER value compared to the control. Blocking MCT4 using the specific inhibitor, α-cyano-4-hydroxycinnamate (CHC), improves the barrier function and alleviates the colitis in DSS mouse models (44). Efforts have been made to develop selective inhibitors against MCT4 like the clinical candidate, AZD0095. This inhibitor showed satisfactory preclinical efficacy for cancer treatment and is on its way to clinical trials (46). Once safety is established, it can be alternatively tested in preclinical and clinical experiments for the treatment of IBD.

### Cellular communication network factor-1

Cellular communication network factor-1 (CCN1) or cysteine-rich angiogenic inducer 61 (Cyr61) is a matricellular protein that is upregulated in colons of CD and UC patients (47). CCN1 induces IL-6 expression as well as other pro-inflammatory genes in macrophages (47, 48) The action of CCN1 on these genes was mediated by α_M_β_2_ integrin and the co-receptor syndecan-4 on macrophages. CCN1 also supports macrophage adhesion *via* these two receptors. These receptors are activated by extracellular matrix (ECM) ligands or cell adhesion and activate the transcription factor NFκB, which then influences the change in pro-inflammatory gene expression (48). CCN1 is restricted to terminally differentiated intestinal cells in the normal colon but is found all over the crypt upon DSS treatment in a mouse colitis model. In the initiation phase of colitis, IL-6 upregulation is independent of CCN1, but later in the repair phase (8 days after DSS administration), IL-6 overexpression is mainly CCN1-dependent (47). Heparan sulfate proteoglycans (HSPGs), which include the co-receptor syndecan-4, are involved in monocyte and fibroblast adhesion to CCN1. Pre-treatment of monocytes with heparin or heparinase I partially inhibited monocyte adhesion to CCN1. Heparin binds CCN1 with high affinity, while heparinase removes cell surface HSPGs like syndecan-4. Concentrations of 0.1–10 µg/ml of heparin reduced monocyte adhesion by approximately 45%, and monoclonal antibodies against integrin α_M_β_2_ also blocked monocyte adhesion to CCN1 (49). As the adhesion of monocytes to α_M_β_2_ integrin and syndecan-4 is linked to signal activation by them, this pathway, specifically syndecan-4, presents a potential therapeutic target for a downstream reduction in IL-6 production. Syndecan-4 is an emerging target for cancer treatment, and several cancer therapeutics have been found to downregulate it like trastuzumab (humanized anti-HER2 monoclonal antibody) and panitumumab (human anti-EGFR monoclonal antibody) (50). Hopefully, once a more specific inhibitor of syndecan-4 is available, it can be multi-purposed for cancer and CD treatment.

### Rhomboid proteases 1 & 2

Another upstream pathway that could be included in the potential target list is the rhomboid proteases iRhom1 and iRhom2 (iRhom 1/2). These are regulators of the membrane trafficking of the transmembrane metalloproteinase, TACE (also known as ADAM17, a disintegrin and metalloproteinase 17). iRhoms form a complex with TACE in the endoplasmic reticulum (ER) and guide TACE trafficking from the ER to the cell surface *via* the Golgi apparatus. In Golgi, the pro-domain is removed, and the proteolytic potential of TACE is released. Ablation of iRhoms retains TACE in ER and impairs its maturation. TACE is also involved in the shedding of many inflammatory cytokines and their receptors including IL-6R, which mediates the trans-signaling on gp130-expressing cells (51). TACE membrane trafficking increases in macrophages during *Mycobacterium avium paratuberculosis* (MAP) infection and in IBD, which results in an upregulation of pro-inflammatory cytokine release (51, 52). iRhom1 and iRhom2 are to a large extent redundant and must be abolished together to achieve an effect on TACE membrane trafficking (51). SiRNA-mediated silencing of iRhom 1/2 in MAP-infected macrophages reduced the production and membrane trafficking of TACE and further decreased the expression of pro-inflammatory cytokines like TNFα (52). Targeting iRhoms would also reduce the shedding of IL-6R from MAP-infected macrophages, abolishing the detrimental increase of IL-6 trans-signaling during MAP-infection. Pharmaceutical targeting of TACE through iRhom 1/2 is an emerging need for many inflammatory disorders like RA, atherosclerosis, Alzheimer’s disease, and IBD (51). Also, further experimentations are needed to identify off-targets and side effects of reducing TACE membrane trafficking.

## The role and interplay of *M. avium paratuberculosis* infection and IL-6 in CD therapy

The etiology of CD is a complex interplay between host genetics, the immune response, and environmental and microbial factors. Some microbes have the potential to stimulate an immune response in genetically susceptible individuals and initiate a chronic inflammatory response in the intestine manifested as CD symptoms (53). Among many debated microbial agents linked to CD, MAP has been heavily studied in clinical samples and found to be closely associated with CD (3, 54). Viable MAP is specifically detected in the blood of more than 50% of CD patients as found in separate studies (3, 55). This bacterium was first studied in mammal ruminants (like cows, sheep, goats, and deer) where it causes an inflammatory bowel disease called Johne’s disease (56, 57). In addition to being present in infected animal’s tissues, MAP is released in their feces and milk. Because this microbe is resistant to conventional pasteurization, humans can be exposed to MAP when in contact with the meat or milk of an infected animal (58). MAP is an obligatory intracellular pathogen that can survive in host macrophages by inhibiting the phagosome–lysosome fusion similar to *Mycobacterium tuberculosis* (54). Evidence on MAP association with CD was derived from the specific detection of MAP DNA in the blood and granulomatous tissue of CD patients using PCR specific to the unique MAP genome sequence, IS*900* (59, 60). It was further supported by the identification of serum antibodies against MAP-specific proteins (p35 and p36), RNA, and culturable microbes from the intestinal tissue, milk, and blood of CD patients (3, 54, 60, 61).

MAP has been meticulously studied in bovine hosts. It typically infects the host through the oral route, inhalation, or exchange of body fluids. Once MAP reaches the intestine, it can invade the intestinal tissue through the microfold (M) cells of Peyer’s patches and the iliac epithelial cells. The movement of MAP across the intestinal layer activates the bacterial cell wall protein, fibronectin attachment protein (FAP), which attaches MAP to the luminal surface of intestinal M cells that express the β1 fibronectin receptor. MAP is subsequently translocated to the submucosa, where it is engulfed by macrophages. There, MAP interferes with the maturation of phagolysosomes, allowing the mycobacterium to survive in host macrophages and sustain a persistent infection (56). MAP can further manipulate macrophage survival by activating Notch-1 signaling to inhibit cellular apoptosis and enhance their survival in the intestinal tissue (62). In macrophages, MAP upregulates the expression and release of TNFα that leads to chronic inflammation in the gut and upregulates the expression of other inflammatory cytokines like IL-6 and IL-12 (52, 63). MAP infection also reduces TGFβ signaling needed for immunosuppression, which allows for tissue damage healing (52). It is strongly supported that this microbial mechanism of establishing a long-standing infection in intestinal macrophages leads to the chronic intestinal inflammation seen in CD.

MAP-targeting antibiotic combinations have shown clinical efficacy in several studies (58). Most notably, a triple antibiotic treatment that comprises clarithromycin (CLA), clofazimine (CLO), and rifabutin (RIF) was evaluated *in vitro* for efficacy against MAP infection (64, 65). This triple therapy reduces NFκB signaling and T-cell proliferation, demonstrating intrinsic anti-inflammatory properties in addition to the antimicrobial ones (66). This treatment has proved efficacy in a clinical trial on active CD patients where clinical response and remission were improved by RHB-104 treatment compared to the placebo group. Notably, the enhanced response with this therapy was more pronounced when combined with other conventional CD treatments like anti-TNFα (67).

IL-6, like other pro-inflammatory cytokines, is upregulated during MAP infection in CD patients. IL-6 serum levels in MAP-positive CD patients were determined as 1.72 ± 1.65 ng/ml, while in MAP-negative patients, it was 0.82 ± 0.74 ng/ml (7). Unlike TNFα, which is needed for the elimination of infectious agents, treatment of MAP-infected macrophages with recombinant IL-6 (rIL-6) increased the survival of MAP in macrophages. This points to IL-6 as a unique and better target for MAP-positive CD patients than conventional targeting of TNFα (63). A combination of anti-MAP and IL-6-targeted therapies could provide hope for CD patients with an underlying MAP infection.

## Conclusion

IL-6 is a versatile cytokine with various functions throughout the body and a more targeted and specialized influence on the intestinal mucosa. It plays a central role in the pathogenesis of CD and other inflammatory disorders. The signaling of IL-6 is of particular interest because it comprises a complex network of multiple upstream regulators, multiple modes of activation, and a variety of downstream signaling pathways activated in target cells. This complexity provides a multitude of targets for fine-tuning the concentration of released IL-6 or the extent of signaling pathway activation in a disease context. In light of this complexity, IL-6 plays a dual role in the intestine, and aberrations in IL-6 concentration and signaling can participate in the shift between homeostasis and chronic inflammation. Substantial efforts have been made to target the IL-6 pathway for the treatment of inflammatory disorders. More trials and efforts should include patients with IBD and CD specifically, as the current approaches are becoming less effective. Regulating IL-6 release and signaling could be the key to providing a healing window for a chronically inflamed intestine. Differentially targeting the trans-signaling pathway of IL-6 could be the key to finding the balance needed to restore intestinal health in patients with CD and other inflammatory disorders.

## Author contributions

AA: Conceptualization, Methodology, Writing – original draft, Writing – review & editing. SN: Conceptualization, Funding acquisition, Methodology, Project administration, Resources, Writing – review & editing.
